# Randomized controlled trial of live lactobacillus acidophilus plus bifidobacterium bifidum in prophylaxis of diarrhea during radiotherapy in cervical cancer patients

**DOI:** 10.1186/1748-717X-5-31

**Published:** 2010-05-05

**Authors:** Imjai Chitapanarux, Taned Chitapanarux, Patrinee Traisathit, Sudkaneung Kudumpee, Ekkasit Tharavichitkul, Vicharn Lorvidhaya

**Affiliations:** 1 Department of Radiology, Faculty of Medicine, Chiang Mai University, Chiang Mai, Thailand; 2 Department of Medicine, Faculty of Medicine, Chiang Mai University, Chiang Mai, Thailand; 3 Department of Statistics, Faculty of Science, Chiang Mai University, Chiang Mai, Thailand

## Abstract

**Background:**

Radiation-induced diarrhea is frequently observed during pelvic radiotherapy. This study was performed to determine the ability of a probiotic containing live lactobacillus acidophilus plus bifidobacterium bifidum to reduce the incidence of radiation-induced diarrhea in locally advanced cervical cancer patients.

**Methods:**

Patients who were undergoing pelvic radiotherapy concurrent with weekly cisplatin were randomly assigned to a study drug or placebo, in a double-blind study. Diarrhea was graded weekly according the Common Toxicity Criteria (CTC) system. Stool consistency and white and red blood cell count in stool were also assessed. The primary endpoint was to reduce the incidence of diarrhea, defined by a CTC grade 2 or more, and the need for anti-diarrheal medication.

**Results:**

A total of 63 patients were enrolled. Grade 2 -3 diarrhea was observed in 45% of the placebo group (n = 31) and 9% of the study drug group (n = 32) (p = 0.002). Anti-diarrheal medication use was significantly reduced in the placebo group (p = 0.03). The patients in the study drug group had a significantly improved stool consistency (p < 0.001).

**Conclusions:**

Live lactobacillus acidophilus plus bifidobacterium bifidum reduced the incidence of radiation-induced diarrhea and the need for anti-diarrheal medication and had a significant benefits on stool consistency.

## Background

Radical radiation therapy to pelvic malignancy carries a risk of complications to normal tissues around the tumor. Acute complications affecting the gastrointestinal tract occur in approximately 80% of patients, but they are usually mild and only rarely affect the treatment planning [1]. One of the most common acute complications of pelvic radiotherapy is acute inflammatory change in the small intestine leading to gastrointestinal symptoms during treatment because healthy bowel tissue is encompassed in the radiation field. Acute symptoms include diarrhea, abdominal pain, tenesmus and nausea, usually starting during the second or third week of radiotherapy [1,2]. Because of this the incidence of malnutrition in patients who receive pelvic radiotherapy is 11-33% and up to 83% of patients lose weight during treatment [3]. The development of late gastrointestinal symptoms following pelvic radiotherapy is not entirely dose related, but depends on a complex interaction between physical, patient-related, treatment-related, and genetic factors that is not well understood [4]; Andreyev et al. have found that in approximately one-quarter of patients referred with gastrointestinal symptoms after radiotherapy the symptoms are unrelated to the radiotherapy itself [5]. However 5-10% of patients who have acute gastrointestinal tract complications during radiotherapy go on to suffer late serious gastrointestinal complications [6-8]. These include bowel obstruction, fistulation and intractable bleeding. The severity of acute bowel toxicity may predetermine the degree of chronic bowel changes [9]. Therefore early intervention to prevent or reduce acute toxicity may also have long term benefits.

The intestinal mucosa depends upon the bacterial flora of the gut and the luminal contents for part of its own supply of nutrients. Radiation creates changes in bacterial flora, the vascular permeability of the mucosal cells and in intestinal motility [9,10]. The ingestion of lactic acid bacteria has been extensively investigated as a beneficial dietary adjunct for gastrointestinal disorders in humans and animals. Lactobacillus has been suggested for the prevention and treatment of diarrhea induced by E. Coli, salmonella or shigella [11]. In vitro studies have indicated that part of this effect may be due to lactobacillus strains [12-15]. Since dysbiosis of the intestinal flora may be a promoting factor in radiotherapy-related intestinal problems, lactobacilli could be used to achieve a more balance micro-flora during the treatment. Mc Gough et al. [3] reviewed the original studies in the management of gastrointestinal tract side effects in patients undergoing pelvic radiotherapy and found that low-fat diets, probiotic supplementation and an elemental diet may be beneficial in preventing symptoms. The objective of our study was to test the efficacy of lactobacillus acidophilus plus bifidobacterium bifidum in reducing the incidence and severity of diarrhea during pelvic radiotherapy.

## Patients and methods

### Study design

This was a prospective, randomized, double-blind, placebo-controlled study. Patients diagnosed with locally advanced cervical cancer and planned to receive concurrent chemoradiotherapy with weekly cisplatin, were randomly assigned to receive either lactobacillus acidophilus plus bifidobacterium bifidum (Infloran^®^) or placebo capsules containing magnesium stearate, talc, and purified water. The placebo was the same size and color as the study drug. Pre-packaged (blinded) study medication differing solely in the patient numbers on the medication package was provided by the sponser. The study drug, Infloran, is manufactured by Laboratio Farmaceutico SIT, Mede, Italy. One capsule (250 mg) contains an oral preparation of a minimum of 1000 million of lactobacillus acidophilus viv. Lyophilisat and minimum of 1000 million of bifidobacterium bifidum viv. Lyophilisat

Patients were stratified by age, stage, and whole pelvis radiotherapy technique. Patients were randomly assigned in a double blind fashion to receive study drug or placebo in a 1: 1 ratio. The study protocol was submitted to the ethical review board of Faculty of Medicine, Chiang Mai University and written informed consent was obtained from each patient.

### Patients

Patients aged at least 18 and not more than 65 years old, with FIGO stage IIB-IIIB squamous cell carcinoma of cervix, who were planned to receive the standard treatment for locally advanced cervical cancer of external beam whole pelvis radiotherapy and brachytherapy plus weekly cisplatin 40 mg/m^2^, with ECOG performance status 0-1 and negative anti-HIV were included. Exclusion criteria were; past history of pelvic radiotherapy or abdominal surgery and diarrhea before the beginning of this study. Patients who had any gastrointestinal disease, pregnant and lactating were also excluded from the study.

### Treatment

After stratification patients were randomly assigned to receive 2 × 10^9 ^units of a lactobacillus acidophilus plus bifidobacterium bifidum (equivalent to 2 capsules) two times a day before meals (morning and evening), beginning 7 days before starting radiotherapy and continuing everyday during radiotherapy. In the other control group, an the identical-appearing placebo was administered in the same schedule. Neither the patient nor the treating physician knew if the patient was on the study drug or placebo. Patients were given standard dietry recommendations for radiation thereapy and in addition all yogurt and other dairy foods produced by fermentation were forbidden. All subjects were scheduled for external pelvic radiotherapy with a dose 200 cGy per fraction, five fractions per week. The superior border of fields were at the L4-L5 junction. The inferior border of fields were at the bottom of obturator foramen or 2 centimeters lower than the lowest margin of tumor. The lateral borders were 2 centimeters beyond each pelvic brim. After 4000 cGy, a midline block was inserted and radiation continued to 5000 cGy. Thereafter the field was reduced to treat only both parametria to a total dose of 5600 cGy. Every patient also received four insertions of brachytherapy with Iridium-192 for 700 cGy per fraction. All patients received weekly cisplatin 40 mg/m^2 ^for 6 weeks during radiotherapy.

### Monitoring and laboratory investigations

Patients were evaluated weekly for the severity of diarrhea according to the National Cancer Institute Common Toxicity Criteria; NCI CTC version 2.0 (grade 0 = none; grade 1 = increase of < 4 stools/day over pre-treatment; grade 2 = increase of 4-6 stools/day, or nocturnal stools; grade 3 = increase of ≥ 7 stools/day or incontinence or need for parenteral support for dehydration; grade 4 = physiologic consequences requiring intensive care, or hemodynamic collapse). The characteristics of the stool, the presence of white or red blood cells in the stool, the use of anti-diarrheal medication and the patient's weight were also recorded weekly. Stool consistency was objectively defined by the technician in the laboratory and cell counts were analyzed by fresh stool examination (wet mount). Hematological toxicities were assessed weekly using the NCI CTC v.2.0. Since the bacilli contained in the study drug are harmless saprophytes, no adverse effects can result from their administration, however, an adverse event or adverse drug reaction was recorded in each week of treatment. No major adverse events owing to probiotic supplemaentation were reported in any study.

Patients were required to return their bottles of study medication (study drug or placebo) weekly and the number of capsules returned was documented. Patients who took < 80% of the medication were considered non-compliant, but were included in the intent-to-treat analysis. Concomitant medications were recorded. Patients who requested anti-diarrheal medication were provided with this by the researcher immediately and recorded with the patient's concomitant medication record. Only Loperamide (2 mg) was used. Adverse events were recorded weekly.

### Statistical Analysis

Continuous variables were described as median and were compared using the Mann-Whitney test. Categorical variables were described as percentage and were compared using the Chi-square or Fisher's tests. The p-values reported are two-tailed and an alpha level of 0.05 was used to assess statistical significance. Sample size calculation was calculated by the formula: n = 2 (Z_α/2 _+ Z_β_)^2 ^σ^2^/(X_1_-X_2_): n = 29 each group.

Statistical analyses were performed using SPSS statistical software (version 11.5, SPSS Inc., 444 N. Michigan, Chicago, Illinois, USA).

## Results

**Figure 1:**
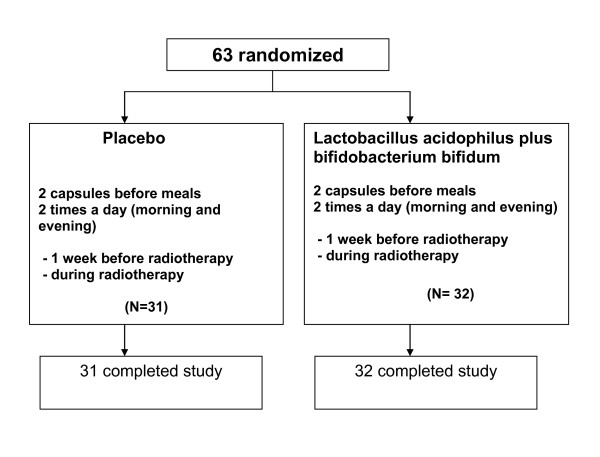
**Trial profile**.

Between January 2007 and April 2009, sixty-three patients were randomly allocated to receive lactobacillus acidophilus plus bifidobacterium bifidum (Infloran^®^) in 32 patients or a placebo in 31 patients (Fig 1). All 63 patients were eligible and assessable. All patients had undergone concurrent chemoradiotherapy. Age, stage of disease, performance status, and whole pelvis radiotherapy technique did not show any difference between the two groups. Baseline characteristics are shown in Table 1. During irradiation, diarrhea occurred in all patients. In the 31 eligible patients who received pelvic radiotherapy and placebo, rates of Grades 1, 2, and 3 diarrhea during treatment were 55, 42, and 3%, respectively. For the 32 eligible patients who received radiotherapy and lactobacillus acidophilus plus bifidobacterium bifidum, the overall rates of grades 1, 2, and 3 diarrhea were 91, 9, and 0%, respectively. The difference in the severity of diarrhea was significant p = 0.002 (Table 2). The patients who received lactobacillus acidophilus plus bifidobacterium bifidum also had a significantly improved stool consistency (p < 0.001) (Table 2). The prevalence of formed, soft, and liquid stool was 0%, 35% and 65%, respectively in placebo group. In contrast, in the study drug group the prevalence of formed, soft, and liquid stool was 3%, 78% and 19%, respectively. However white and red blood cell counts in patients' stool did not differ between the two groups. The severity of radiation-induced diarrhea is illustrated by 32% of patients in the placebo group needing anti-diarrheal medication, as against 9% of patients in the study drug group (p = 0.03). The median overall treatment time and median weight change from the beginning to the last treatment did not differ between groups (Table 2). There were no adverse events attributable to the study drug.

**Table 1:** Baseline characteristics

Characteristics	Placebo group (n = 31)	Lactobacillus acidophilus plus bifidobacterium bifidum (Infloran^®^) group (n = 32)	p-value
Median age (year)	52	47	0.146^§^

Stage of cervical cancer			0.80^¶^

IIB, n (%)	18 (58.1)	17 (53.1)	

IIIB, n (%)	13 (41.9)	15 (46.9)	

ECOG performance status			0.08^¶^

0, n (%)	29 (93.5)	24 (75.0)	

1, n (%)	2 (6.5)	8 (25.0)	

Whole pelvis Radiotherapy technique			0.60^¶^

AP-PA technique, n (%)	22 (71.0)	20 (62.5)	

4-field box technique, n (%)	9 (29.0)	12 (37.5)	

Tests: ^¶ ^Fisher's exact test; ^§ ^Mann-Whitney.

**Table 2:** Treatment outcome

Characteristics	Placebo group n = 31 no. (%)	Study group n = 32 no. (%)	p-value
Median overall treatment time (days)	51	48	0.05^§^

Median weight change from the beginning to the last treatment (kg)	-2.5	-2.4	0.66^§^

Diarrhea			0.002^Θ^

Grade I	17 (55)	29 (91)	

Grade II, III	14 (45)	3 (9)	

Anti diarrhea drug used			0.03^¶^

No	21(68)	29(91)	

Yes	10(32)	3(9)	

Stool			<0.001^Θ^

Soft, Form	11(35)	26 (81)	

Loose	20 (65)	6 (19)	

White blood cell in stool			0.08^¶^

No	24 (77)	30 (94)	

Yes	7 (23)	2 (6)	

Red blood cell in stool			0.11^¶^

No	28 (90)	32(100)	

Yes	3 (10)	0(0)	

Tests: ^¶ ^Fisher's exact test; ^§ ^Mann-Whitney; ^Θ ^Chi-Square.

## Discussion

The present study shows that during pelvic radiotherapy for cervical cancer diarrhea occurred in 100% of our patients. However this side effect was rarely severe, the median maximum severity of diarrhea was only grade 1. In Baughan's study [16], median maximum severity of diarrhea was also grade 1. Although diarrhea usually accompanies rectal mucosal lesions, some authors believe that it is caused by radiation injury to the small intestine. Bile acid malabsorption and bacterial contamination by an aerobic and anaerobic bacteria are common causes of diarrhea after the radiation treatment of gynecological cancer. Maintaining intestinal integrity during radiotherapy significantly influences the quality of life of patients. Usually severe radiation-induced diarrhea is treated by medication but it may lead to an interruption in treatment. Probiotic nutritional intervention before and during radiotherapy may induce a radio-protective effect for healthy tissues, the mechanism of which is not clear. It is assumed that probiotics may improve the immune status of the gut [17]. Also the growth of probiotics may interfere with the growth of pathogenic bacteria because they compete with pathogenic bacteria for binding sites on epithelial cells [18]. Previous studies of Lactobillus bacteria for radiation induced diarrhea have produced mixed results. Two randomly controlled trials were negative, failing to identify significant improvements in chronic bowel symptoms in patients randomized to the study drug [19,20]. Other randomized studies using probiotics during pelvic radiotherapy for diarrhea prophylaxis have demonstrated a decrease in the mean number of bowel movements and the incidence of diarrhea [21-23]. Similar results have been obtained in our study; the group receiving lactobacillus acidophilus plus bifidobacterium bifidum experienced less grade 2 or 3 diarrhea and required less anti-diarrheal medication compared to the placebo group. Stool consistency was also better in the treatment group than the study group, but no difference in white and red cell count was seen. One possibly significant difference of our study to those with negative symptomatic results is the use of a combination of bacterial strains (lactobacillus acidophilus and bididobacterium bifidum) rather than just a single strain. Delia et al. [22] suggested that the use of several selected strains could enhance the competitive interaction with the intestinal flora. Thus the relative success of our study may be due to the strains of bacteria used, the probiotic concentration or the synergistic effect of combining more than one strain. In this study, we did not perform the stool culture, this could be the weakness of the study (it may be the other cause of diarrhea), anyway, we perform the white blood cell count in stool to help us to discriminate the infectious diarrhea. We conclude that we can use live lactobacillus acidophilus plus bifidobacterium bifidum for diarrhea prophylaxis during pelvic radiation therapy with concomitant cisplatin for locally advanced cervical cancer, with favorable reductions in symptoms.

## Competing interests

The authors declare that they have no competing interests.

## Authors' contributions

IC participated in the design of the study, and drafted the manuscript.

TC participated in its design and coordination.

PT performed the statistical analysis.

SK carried out the questionnaires, and stool collection.

ET participated in coordination.

VC conceived of the study.

All authors read and approved the final manuscript.
